# Partial Contribution of Socioeconomic Factors to the Mortality Rate of the Working-Age Population in Russia

**DOI:** 10.3390/healthcare11101507

**Published:** 2023-05-22

**Authors:** Mihajlo Jakovljevic, Olga Kozlova, Maria Makarova, Natalia Neklyudova, Olga Pyshmintseva

**Affiliations:** 1 Institute of Advanced Manufacturing Technologies, Peter the Great St. Petersburg Polytechnic University, 195251 St Petersburg, Russia; 2 Institute of Comparative Economic Studies, Hosei University, Tokyo 194-0298, Japan; 3 Department of Global Health Economics and Policy, Faculty of Medical Sciences, University of Kragujevac, 34000 Kragujevac, Serbia; 4 Institute of Economics, Urals Branch, Russian Academy of Sciences, 620014 Ekaterinburg, Russia

**Keywords:** working-age population, health, mortality, socioeconomic factors, health sector, partial contribution

## Abstract

This study’s relevance lies in the need to assess the role of socioeconomic, medical, and demographic factors on working-age population mortality in Russia. The purpose of this study is to substantiate the methodological tools for the assessment of the partial contribution of the most important factors that determine the dynamics of the mortality of the working-age population. Our hypothesis is that the factors determining the socioeconomic situation in the country affect the level and dynamics of mortality of the working-age population, but to a different extent in each separate period. To analyse the impact of the factors, we used official Rosstat data for the period from 2005 to 2021. We used the data that reflect the dynamics of socioeconomic and demographic indicators, including the dynamics of mortality of the working-age population in Russia as a whole and in its 85 regions. First, we selected 52 indicators of socioeconomic development and then grouped them into four factor blocks (working conditions, health care, life security, living standards). To reduce the level of statistical noise, we carried out a correlation analysis, which allowed us to narrow down the list to 15 key indicators with the strongest association with the mortality rate of the working-age population. The total period of 2005–2021 was divided into five segments of 3–4 years each, characterising the picture of the socioeconomic state of the country during the period under consideration. The socioeconomic approach used in the study made it possible to assess the extent to which the mortality rate was influenced by the indicators adopted for analysis. The results of this study show that over the whole period, life security (48%) and working conditions (29%) contributed most to the level and dynamics of mortality in the working-age population, while factors determining living standards and the state of the healthcare system accounted for much smaller shares (14% and 9%, respectively). The methodological apparatus of this study is based on the application of methods of machine learning and intelligent data analysis, which allowed us to identify the main factors and their share in the total influence on the mortality rate of the working-age population. The results of this study show the need to monitor the impact of socioeconomic factors on the dynamics and mortality rate of the working-age population in order to improve the effectiveness of social programme. When developing and adjusting government programmes to reduce mortality in the working-age population, the degree of influence of these factors should be taken into account.

## 1. Introduction

According to the Russian Labour Code, the working-age population in Russia is defined as men aged 16 to 65 and women aged 16 to 60 (Russian Labour Code [URL]: https://www.consultant.ru/document/cons_doc_LAW_34683/ (accessed on 1 March 2023)). In Russia, despite a 32.4% decrease in mortality in the working-age population in 2021 compared to 2005, there has been an increase in mortality from 2020 due to an increase in the incidence of COVID-19. In 2021, COVID-19 caused 66,500 deaths in the working-age population, or 13.4% (Demographic Yearbook of Russia-2021. URL: https://rosstat.gov.ru/folder/210/document/13207 (accessed on 25 April 2023)) of all deaths in the working-age population. Considering the mortality structure of the working-age population, there was a decrease in all death classes, except neoplasms, other causes, and the COVID-19 class of deaths from 2020. The increase in deaths from other causes was due to an increase in deaths from diseases of the endocrine and nervous system. Although the consumption of strong alcoholic drinks has been decreasing in Russia since 2005, the consumption of beer drinks is increasing, which has a deleterious effect on the growth of obesity-related diseases and diabetes mellitus.

The most urgent problems related to health loss and mortality in the working-age population should be noted.

There is a higher mortality of men compared to women. Note that since 2006, the mortality rate of men of working age has decreased faster than that of women, but still remains high (over 800 deaths per 100,000 of the working-age population). The reduction in male mortality is mainly due to a reduction in preventable deaths from external causes (almost 2.5 times during the analysed period).

A significant contribution of external causes to mortality at a young working age (56% of 15–29 year olds in 2021) is due to low level of self-preservation behaviour, characteristic mainly of young men.

Numerous cases of violations of labour protection requirements by both employers and employees and a high share of those employed in harmful and hazardous working conditions (6.7% of the total employed population in 2021) contribute to injuries in the workplace. Although this has almost halved in 2021 compared to 2005, it remains high. There are more than 1000 fatal accidents at work in Russia each year (Number of sufferers in industrial accident with a disability of one working day or more and fatal accidents//EMISS. URL: https://www.fedstat.ru/indicator/data.do?id=31559 (accessed on 26 April 2023)).

Despite a reduction in mortality among the working-age population from 2005 to 2021, the incidence of socially significant diseases has increased among the general population. The number of illnesses has increased compared to 2005: 7.4% for ‘neoplasms’, 38.4% for ‘respiratory diseases’, 18.7% for ‘endocrine system diseases’, and 32.6% for ‘circulatory system diseases’ (Supplement to Regions of Russia. Socioeconomic Indicators, 2022. URL: https://rosstat.gov.ru/folder/210/document/47652 (accessed on 10 January 2023)).

As a result, the relationship between health, mortality, and socioeconomic factors is an urgent research issue, especially in the context of the ongoing COVID-19 pandemic and the increase in other biomedical (new diseases) and sociopsychological (the level of social stress accumulated in society) threats.

## 2. Literature Review

The literature on the impact of socioeconomic factors on health and mortality takes two main approaches.

One group of authors considers health and mortality as determinants of human quality of life and national economic development. These papers focus on studies of the negative psychosocial consequences of poor quality of life [1], including the effects of social isolation [2,3], self-perception of aging [4], and subjective wellbeing [5]. There are researchers that studied the state of health and dynamics of population mortality as factors of differentiation of economic development of territories [6,7] and estimated the level of morbidity and mortality [8,9]. The problems of declining health of the population are studied separately, which determines the need to develop new approaches to the implementation of public health policy [10].

The second group seeks to explain how health is formed and on what factors it depends; in other words, they consider health as an explanatory variable. Among the predictors of health, researchers have studied the following.

Firza and Monaco studied how lifestyle determines population health compared to sociodemographic characteristics [11]; Roberts et al. analysed how emotional state affects the likelihood of injury and back pain [12], while Moore S. et al. studied how emotional state affects gaming disorder [13]. Socioeconomic inequalities and other institutional barriers that determine the availability of health care were researched by Wilson L. et al. [14] and Østergaard M.L.D. et al. [15], while Sana Sh. et al. and Camp J. et al. studied the demand for health care [16,17]. Sun Zh. et al. tried to understand how risk levels in the wider community determine the incidence of diabetes and high blood pressure [18].

There are also studies of how behavioural activities shape health or its loss in certain sociodemographic groups, including how eating behaviour and food addictions shape the health quality of adolescents [19]; how behavioural activity and living environment influence the health status and morbidity of rural residents [20]; and how the physical activity of adolescents determines their wellbeing [21]. A cohort of studies investigated social determinants of health [22], health literacy, and behavioural health factors in adults [23], as well as differences in health status between urban and rural residents [24]. Schmits E. and Glowacz G. highlighted the negative impact of isolation during the coronary crisis on increased alcohol consumption and increased anxiety and depression [25]. Schultz-Knudsen M. and Janbek J. studied the role of religion in health promotion [26]. Factors of physical (natural) and social environments determining the health of the population have attracted the attention of researchers [20], including audiological risk factors [27]. Bright S. J. et al. discussed genetic predisposition to certain types of diseases [28]. Dayyab F. et al. studied the role of geographical factors [29], and Anam S. and Shar N. A. studied the role of climatic factors [30]. The role of socioeconomic factors in population health, disease transmission, and suicide were also highlighted [31,32]. Nurhayati A. et al. outlined the relationship between working conditions and working environment and occupational disease incidence [33]. Orlova E. insists that investing in workers’ health ensures productivity growth [34]. The effectiveness of the health care system and health service coverage are discussed in papers of Jakovljevic M. et al. [35] and Ranabhat C.L. et al. [36]. There are also studies of the relationship between the quality of the health care system and mortality of the population [37,38] and an assessment of socioeconomic damage from the mortality of the working-age population [39]. The results of the literature review indicate a significant relevance to the research of the problems of factor influence and its consequences on the health status and mortality dynamics of the population.

## 3. Data and Methods

### 3.1. Data

Given the close relationship between such phenomena as health and mortality, the authors conclude that an integral measure of the health of the working-age population can be its mortality rate, calculated per 100,000 people in this sociodemographic group.

Russia is a vast country with significant differentiation of natural–climatic, socioeconomic, medical–demographic, cultural, and other conditions of population reproduction. This determines the differentiation of the level of health of the population in different regions and provides rich statistical material for the study of health predictors.

The sources of data for the study are data from the Federal State Statistics Service of Russia, describing the economic and social development parameters of Russia and its regions for the period 2005–2021 (URL: https://rosstat.gov.ru/folder/210/document/13204 (accessed on 25 April 2023). The unit of analysis is the Russian regions. According to the administrative-territorial division of the Russian Federation for 2021, it included 85 regions (subjects of the Russian Federation). Thus, the sample was comprised of 20,400 observations on the basis of statistics of 15 indicators (the rationale for their selection is given below) in 85 regions over 16 years.

Based on a wide range of previous studies on similar topics and taking into account the availability of statistical data in the context of Russian regions, we selected 52 indicators of socioeconomic development that may have an impact on the mortality rate of the working-age population. At the next stage, a pairwise correlation analysis was carried out between the target working-age population mortality indicator and each of the 52 indicators characterising the socioeconomic development of regions. The final sample included indicators with a correlation coefficient of more than 5%, i.e., statistically significant for explaining the variation of the dependent variable.

Then, we excluded the indicators that have significant variation by regions of Russia, which, even if there is a significant correlation, do not allow us to formulate an adequate hypothesis about the impact of this factor on the mortality rate of the working-age population in the whole country. For example, according to this logic, indicators characterising the environmental situation in the regions were excluded, because regions with unfavourable environmental situation can have both extremely high and very low mortality rates of the working-age population, which is due firstly to the poor quality of measurements and secondly to their indirect and delayed impact on health (Table 1).

Table 1 shows that the highest impact on mortality in the working-age population is most closely correlated with indicators characterising the healthcare system, working conditions, and the general level of life safety in society.

It should be noted that the three indicators are negatively correlated with the working-age mortality rate. Thus, according to our calculations, a lower level of unemployment is associated with a higher mortality rate of the working-age population. This could be explained by the poor quality of working conditions in the workplace, which is confirmed by the statistics at the beginning of the article about the large proportion of jobs in the Russian economy having difficult and dangerous working conditions. The same is confirmed by the negative correlation with the tertiary education of the workforce: the higher the share of the workforce with tertiary education (which allows for more modern and health-promoting jobs), the lower the mortality rate of the working-age population. The rather high but negative correlation between working-age mortality and paved road density suggests that poor pavement quality causes many road crashes with quite serious consequences, up to fatalities.

At the same time, a number of indicators were excluded. These were indicators for which a relatively significant correlation was found, but the sign of the correlation coefficient did not lend itself to a logical explanation. For example, the working-age mortality rate and the environmental indicators have an inverse correlation, which contradicts the logic and data of the available studies. Therefore, we assume that the environmental factor is not an explanatory factor in the formation of the working-age mortality rate, or that the relationship is more complex and its inclusion in the analysis requires further research.

As a result of the preliminary data analysis, 15 indicators characterising the level of socioeconomic development of Russian regions and having a statistically significant relationship with the mortality rate of the working-age population were selected. The indicators were grouped into four logical sets (Table 2).

The selection of indicators and their grouping into factor blocks allowed us to formulate the hypothesis that the factors determining the socioeconomic situation in the country do affect the level and dynamics of mortality in the working-age population, but to a different extent in each separate period.

According to the WHO, health is a state of complete physical, mental, and social well-being, and not merely the absence of disease or infirmity (URL: https://www.who.int/about/frequently-asked-questions (accessed on 25 April 2023). Without addressing the issues of genetic predisposition and environmental conditions, we talk about the factor influence of social and economic parameters of life activity on mortality in the working-age population and assume that their cumulative influence is 100%. Firstly, it makes it possible to abstract away from many related processes and connections. Secondly, it allows the effect of this or that factor to be revealed in its pure form. Of course, the 15 selected indicators are not exhaustive for assessing factor influence, but we can assume that they indirectly aggregate the influence of other explanatory variables.

### 3.2. Methods

To calculate the partial contribution of socioeconomic factors to the mortality rate of the working-age population, we used machine learning methods related to data mining.

In our particular case, we were trying to solve the problem of regression reconstruction. Correct feature selection and feature engineering are important considerations in solving this problem. While feature engineering relies more on intuition and expert knowledge, there are a large number of ready-made algorithms for feature selection. So-called “tree” algorithms allow for the calculation of feature informativeness. All other methods are based on an efficient enumeration of subsets of features to find the best subset on which the constructed model gives the best quality. One such enumeration algorithm is the recursive feature elimination algorithm, available in the Scikit-Learn library0 (URL: https://scikit-learn.org/stable/auto_examples/inspection/plot_permutation_importance.html (accessed on 25 April 2023) for the Python programming language.

To successfully apply machine learning techniques, the data were reduced to a comparable form. Once the data were processed, we moved on to the building of the model. To select a model, we used the following methods: the method of least squares; the random forest method; the logistic regression method; the support vector method; the nearest neighbour method.

**Figure 1:**
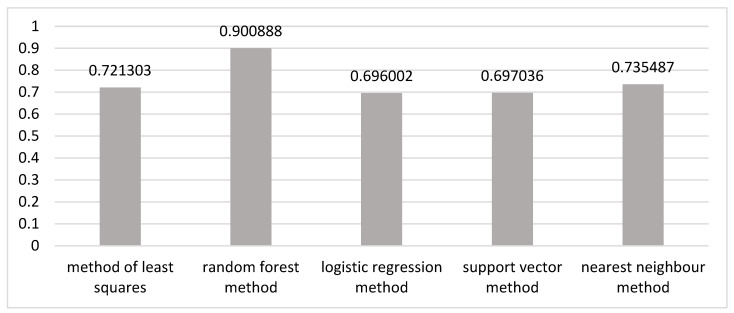
Coefficients of determination for different methods of modelling the partial contribution of socioeconomic development factors to working-age mortality.

We divided the original data sample for 2005–2021 into a test and a training sample, predicted the output variable *y* using models, and compared it with already known data. We obtained the following coefficients of determination (Figure 1), on the basis of which the optimal model was selected.

Figure 1 shows that the “random forest” method performed best in modelling the partial contribution, as its coefficient of determination was higher than the others. This method was used in our study.

A similar procedure was used to select the optimal model for each period analysed. The random forest method was found to be the best for all periods.

The partial contribution was derived from a regression model of the 15 selected indicators, based on random forest, which showed the best predictive power among several other algorithms of this kind. Each individual decision tree was generated using MDI (mean decrease in impurity) indicator selection metrics to calculate the importance of each attribute, or in other words, the proportion of each variable’s contribution to the forecast of the target working-age population mortality rate. The Scikit-Learn library automatically calculates a relevance score for each attribute during the training phase. The partial contribution of socioeconomic indicators into the index of mortality in the working-age population was calculated for each of the indicators presented in Table 1. Then, the contribution values of individual indicators were summed up by factor blocks “working conditions”, “state of the healthcare system”, “life safety”, and “living standards”. In this article, we provide the analysis of the partial contribution predominantly by factor blocks, highlighting individual significant indicators. In the course of this study, the assumption was applied that the total contribution of the analysed factors to the mortality rate of the working-age population is 100%.

The analysis of partial contributions was predominantly by factor block, highlighting individual indicators of significance. In the analytical part of this article, the partial contribution is considered not only by factor blocks, but also by highlighting the indicators that have the greatest impact on the dynamics of the mortality indicator.

We separately studied the partial influence of socioeconomic factors on the mortality of the working-age population in different periods of the social development of Russia and identified the following periods.

- 2005–2008: precrisis period (the end of 2008—global financial crisis);
- 2009–2011: crisis (consequences of the global financial crisis);
- 2012–2014: postcrisis (recovery period after the global financial crisis);
- 2015–2018: sanctions period (after the annexation of the Crimea to Russia);
- 2019–2021: period of increasing sanctions and the COVID-19 pandemic.

## 4. Results

The results of the study are based on the statistical base of indicators of socioeconomic development of Russian regions contained in the statistical collections for 2006–2022, available on the website of the Federal State Statistics Service: https://rosstat.gov.ru/ (accessed on 25 April 2023).

**Figure 2:**
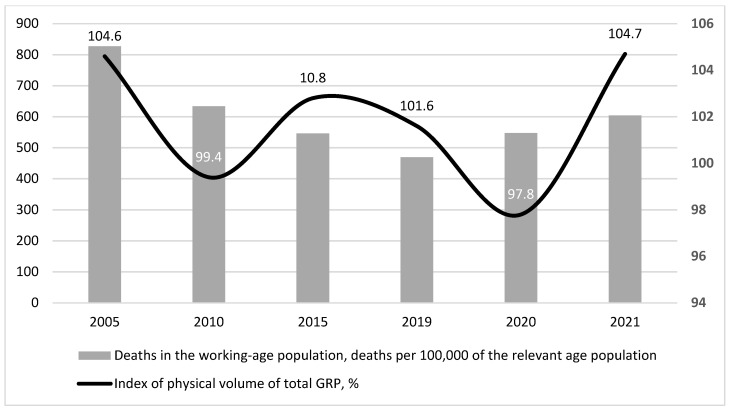
Dynamics of working-age population mortality rate and total GRP in Russia in 2005–2021. Compiled from: Regions of Russia. Socioeconomic indicators, 2022. [Electronic resource]: https://rosstat.gov.ru/folder/210/document/13204 (accessed on 15 January 2023).

Analysing the mortality dynamics of the working-age population in Russia, it should be noted that during the period under consideration (2005–2021), the mortality rate declined from 827.8 deaths per 100,000 people of relevant age in 2005 to 470.0 deaths per 100,000 people of relevant age in 2019. Unfortunately, the new coronavirus pandemic had a negative impact on the mortality rate of working-age people, and by 2021, it had risen to 604.6 deaths per 100,000 people of relevant age (Figure 2).

Figure 2 shows that the mortality rate of the working-age population in Russia responds to the dynamics of total GDP with a certain time lag in the period 2005–2021. In addition, since 2015, an improvement in the overall social and economic situation of the country is characterised by a decrease in the mortality rate of the working population, while a worsening of the economic development indicators is characterised by an increase in the mortality rate of the working population. The exception is probably 2021, when the economic consequences of the new coronavirus pandemic have been overcome, but the demographic consequences (in terms of health deterioration and increased mortality) have not.

**Figure 3:**
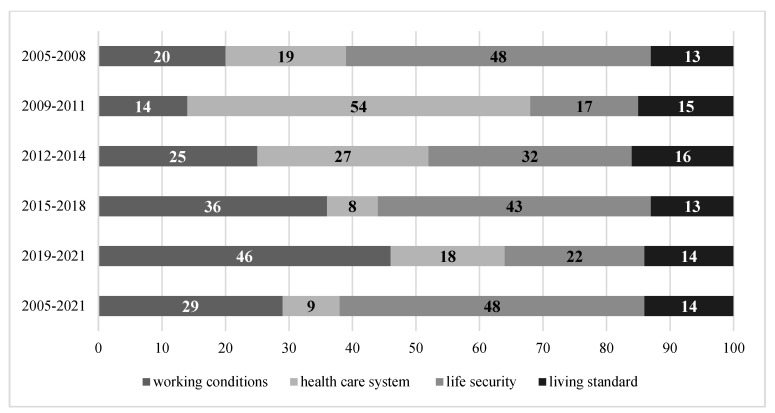
Partial contribution of socioeconomic development factors to mortality of working-age population in Russia in 2005–2021, %.

As noted above, each of the five intervals in the analysis period 2005–2021 had its own specific socioeconomic development and mortality patterns. This allowed us to determine the specifics of the partial contribution of socioeconomic factors in the formation of mortality rates of the working-age population at each of the stages under consideration (Figure 3).

**Figure 4:**
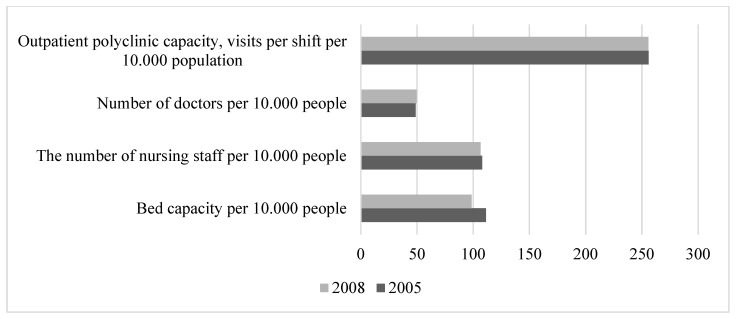
Dynamics of healthcare system indicators in 2005–2008. Compiled from: Regions of Russia. Socioeconomic indicators, 2022. [Electronic resource]: https://rosstat.gov.ru/folder/210/document/13204 (accessed on 15 January 2023).

The period of rather favourable external economic and political conditions in Russia in the years 2005–2008 was characterised by economic and social stability. Compared with the period of previous years, there was an increase in total GDP. Real household incomes increased. This contributed to a decrease in the share of people with incomes below the minimum subsistence level from 17.7% in 2005 to 13.1% in 2008. The unemployment rate fell from 7.2% in 2005 to 6.3% in 2008. Overall, rather favourable economic trends contributed to the reduction in mortality in the active working-age population (Figure 2). At the same time, there was no development in the health sector. The number of beds decreased, but the number of visits to outpatient and polyclinic institutions per shift remained at a rather high level. The number of doctors increased slightly, but the number of nurses decreased (Figure 4). During this period, factors determining life safety (48%), working conditions (20%), and the state of the healthcare system (19%) made the largest contribution to the mortality of the working-age population (Figure 3). This influence was primarily characterised by indicators such as the number of registered crimes (37.5%), the proportion of the working population with a tertiary education (11%), and the number of visits per shift to outpatient clinics (8%).

The increase in economic activity contributed to an increase in employment, including in hazardous, dangerous, and dirty work. In manufacturing, transport, and communications, the share of those employed in harmful and difficult jobs increased from 27% in 2005 to 34% in 2008 (The share of employees working in harmful (or hazardous) working conditions in organisations. URL: https://rosstat.gov.ru/working_conditions (accessed on 6 March 2023)). However, this factor did not have a significant negative impact on the mortality trend (it only accounted for 3% of the total factor effect).

**Figure 5:**
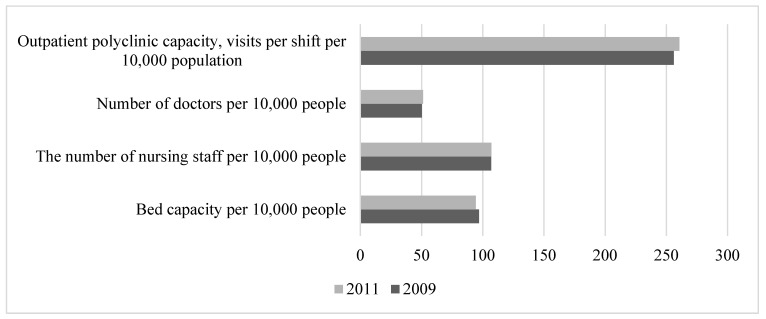
Dynamics of healthcare system indicators in 2009–2011. Compiled from: Regions of Russia. Socioeconomic indicators, 2022. [Electronic resource]: https://rosstat.gov.ru/folder/210/document/13204 (accessed on 15 January 2023).

The period 2009–2011 was characterised by the crisis and postcrisis state of the economy, accompanied by increased social tensions. This period was marked by the adaptation of the population to the realities of the economic crisis. By 2009, real household incomes and employment had fallen, and the unemployment rate had risen to 8.4%. Due to employers’ lack of incentives to take concrete measures to eliminate the impact of harmful, hazardous, and arduous working conditions on workers, the share of jobs with such conditions in the economy remained high, and showed an upward trend (from 37.9% in 2009 to 40.7% in 2011). The government’s anticrisis measures to support businesses and the population had a significant positive impact on the economy and society during this period, helping to stabilise employment and consumer demand. Thanks largely to these measures, the unemployment rate had fallen to 6.6% by the end of the period. In the same period, the reform of cost optimisation in the health sector began, which led to a further reduction in the number of beds and an increase in the number of visits per shift in outpatient clinics (Figure 5).

Despite the economic crisis in the country, the mortality rate of the working-age population has been declining, but at a slower pace. In 2009–2011, the mortality rate of the working-age population decreased by 6.2% to 600.9 deaths per 100,000 persons of relevant age in 2011.

The greatest contribution to the formation of the level and dynamics of mortality of the working-age population was made by factors determining the state of healthcare (54%), life safety (17%), and working conditions (14%). Moreover, the importance of the role of healthcare in shaping the dynamics of mortality of the working-age population increased significantly in comparison with the previous period (by 35 p.p.). 

In our study, the main indicator of the state of healthcare is the number of visits per shift in outpatient clinics, which contributed 45.7% to the mortality rate. 

**Figure 6:**
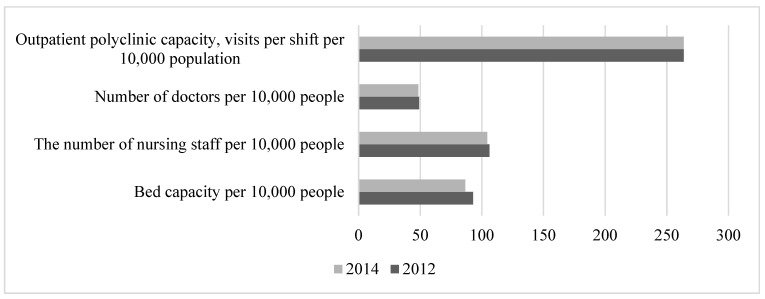
Dynamics of healthcare system indicators in 2012–2014. Compiled from: Regions of Russia. Socioeconomic indicators, 2022. [Electronic resource]: https://rosstat.gov.ru/folder/210/document/13204 (accessed on 15 January 2023).

The period 2012–2014 was characterised by a more difficult foreign policy and foreign economic situation. At the same time, the socioeconomic situation inside the country was more or less favourable, total GRP was growing, and the unemployment rate continued to fall (from 5.5% in 2012 to 5.2% in 2014), which began back in the previous period. The negative processes included further deterioration of working conditions (the share of those employed in harmful, hazardous, and arduous working conditions reached 53% by 2014), as well as the deterioration of the healthcare system. The deterioration in the parameters of the functioning of the healthcare system was associated with a further reduction in bed capacity and a lack of growth in the capacity of outpatient clinics (Figure 6).

Between 2012 and 2014, factors affecting life safety (32%), the state of the healthcare system (27%), working conditions (25%), and standards of living (16%) contributed almost equally to the mortality rate and dynamics of the working-age population (Figure 3).

The leading indicator for reducing mortality among the working-age population was the capacity of outpatient clinics (21.1%). The second leading indicator was the proportion of household expenditure spent on the consumption or purchase of alcoholic beverages (16.5%), followed by the share of people working in hazardous, dangerous, or difficult conditions (16.2%).

In the period of 2015–2018, on the one hand, Russia’s economy was recovering from the crisis and showing growth, but on the other hand, the first sanctions were imposed by Western countries on individuals and companies, which initiated negative processes in the economy due to increasing restrictions on access to foreign markets. During this period, the physical volume index of total GRP averaged around 100% and showed a declining trend. The mortality rate of the working-age population continued to decline during this period: while in 2015, the mortality rate was 546.7 per 100,000 people, by 2018, it had fallen to 482.2 per 100,000 people. At the same time, the average morbidity rate remained stable, at around 780.8 first-time diagnoses per 1000 people. The share of those employed in harmful, hazardous, and difficult working conditions also remained quite high, amounting to 52.7% by 2018. This had a negative impact on population health.

**Figure 7:**
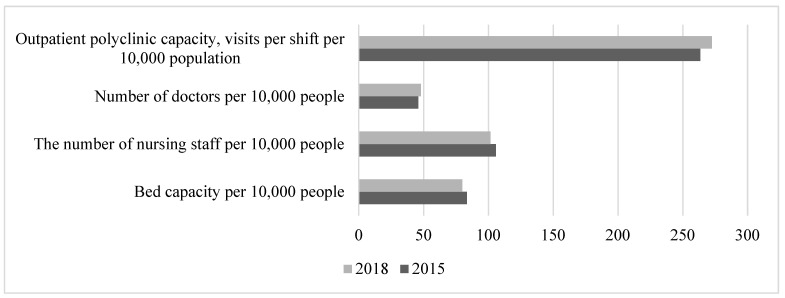
Dynamics of healthcare system indicators in 2015–2018. Compiled from: Regions of Russia. Socioeconomic indicators, 2022. [Electronic resource]: https://rosstat.gov.ru/folder/210/document/13204 (accessed on 15 January 2023).

In the healthcare system, the trend continued towards a change in the priority forms of interaction with the population: the availability of inpatient care was reduced in favour of expanding outpatient care (Figure 7).

During this period, the largest contributors to the working-age mortality rate was made by factors determining working conditions (36%), including the share of people working in hazardous, dangerous, and difficult working conditions (23%); and life safety (43%), including the number of recorded crimes (23%) and the share of household expenditure on alcohol consumption (17%). The contribution of the healthcare system to improving the health and reducing the mortality of the working-age population was only 8%. Thus, during this period, the partial contribution of the healthcare system was the lowest in the whole observation period, and the overwhelming role in reducing the mortality of the working-age population was played by factors reflecting the economic and social wellbeing of the country’s residents.

The period of 2019–2021 in Russia’s socioeconomic development was characterised by the increasing impact of expanding sanctions, the severe consequences of the lockdown resulting from the pandemic of a new coronavirus infection, and consequently, a decline in the physical volume index of the total GRP to negative values. The exception was 2021, when the increase in the index was associated with the beginning of the economic recovery period in the country. At that time, the mortality rate of the working-age population was increasing, primarily due to excess mortality from COVID-19 and the severe restrictions on access to healthcare due to the conversion of health facilities to treat COVID-19 patients and an acute shortage of health personnel. The mortality rate of the working-age population increased from 470 per 100,000 people in 2019 to 604.6 per 100,000 people in 2021. During the same period, the morbidity rate also increased significantly, from 780.2 first-time diagnosed cases in 2019 to 857.1 similar cases in 2021.

**Figure 8:**
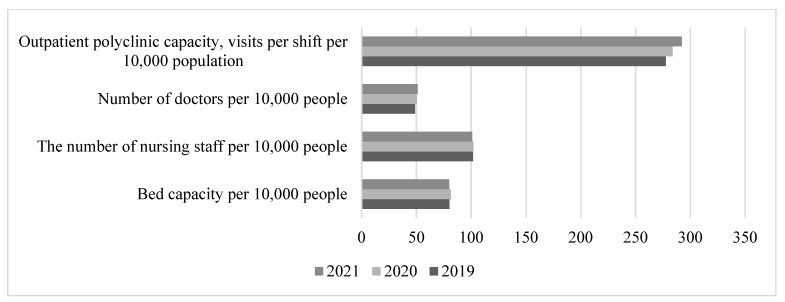
Dynamics of healthcare system indicators in 2019–2021. Compiled from: Regions of Russia. Socioeconomic indicators, 2022. [Electronic resource]: https://rosstat.gov.ru/folder/210/document/13204 (accessed on 15 January 2023).

The healthcare system during this period fell on hard times. On the one hand, the new coronavirus pandemic brought serious changes to its work, requiring the opening of additional inpatient beds and an increase in the number of doctors and nursing staff to combat the new coronavirus infection. On the other hand, activities continued to optimise areas of medical care for the population, and after the decline of COVID-19, the dynamics of the beds and nursing staff returned to their previous downward trajectory (Figure 8).

However, in terms of the quality of employment, there were some improvements in this period: the share of those employed in hazardous, dangerous, and difficult working conditions fell from 53.8% in 2019 to 52.6% in 2021, and the unemployment rate remained stable, at 4.6–4.8%, with occasional spikes in the lockdown months.

Thus, it was the pandemic and its medicodemographic and socioeconomic consequences that determined the mortality dynamics of the working-age population in 2019–2021. This fact is also reflected in the partial contribution of individual socioeconomic factors. In first place remains one of the leading indicators—the share of those employed in harmful, hazardous, and difficult working conditions (34%), which is also due to the suspension of occupational examinations and preventive measures for such categories of the employed population. In second place, there is the number of recorded crimes, with a contribution of 15%. It should be noted that this indicator took the first and the second places throughout the analysed period, but in 2019–2021, it significantly lost its importance (for comparison, in 2005–2008, its contribution was 37%). The third place is occupied by the capacity of outpatient clinics, which contributed 12%, while in the previous period of 2015–2018, it was only 4%.

Thus, working conditions (46%) and life safety (22%) made the largest partial contribution to mortality in the working-age population between 2019 and 2021. The contribution of the healthcare system was 18%, with an increase of 10 p.p. over the previous period. The contribution of standards of living amounted to 14%.

**Figure 9:**
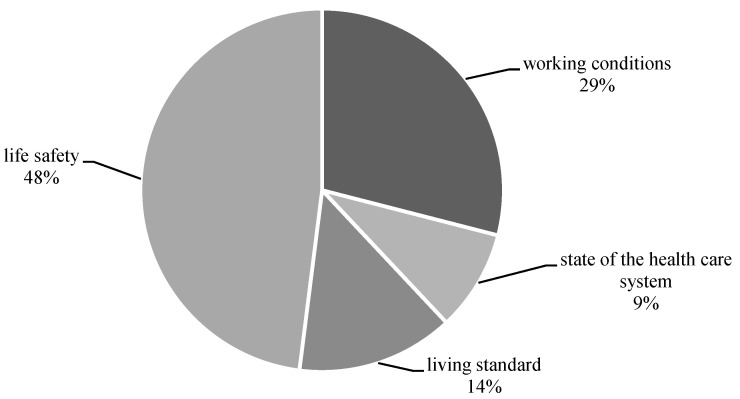
Partial contribution of socioeconomic factors to the mortality rate of the working-age population in Russia as a whole over the period 2005–2021, %.

Over the whole analysed period, the greatest factor contribution to the level and dynamics of the mortality rate of the working-age population was made by the factors of life safety (48%) and working conditions (29%), while factors determining standards of living and the state of the health care system accounted for a much smaller share (14% and 9%, respectively) (Figure 9).

The leading partial contributors are factors of violent and preventable mortality, such as the number of recorded crimes (41%); and factors forming working conditions, such as the share of the labour force with tertiary education (14%) and the share of the workforce working in hazardous, dangerous, and difficult working conditions (12%), which determine the long-term adverse health effects for the working-age population.

A positive trend of mortality decline in the working-age population was recorded during 2005–2021, from 827.8 per 100,000 people in 2005 to 470.0 per 100,000 people in 2019 (the last prepandemic year), but during the next two years, an increase to 604.6 in 2021 was recorded (Figure 2. At the same time, despite the decline in mortality among the working-age population, the morbidity rate increased throughout the analysed period, from 744.4 first-time diagnoses per 1000 people in 2005 to 857.1 in 2021.

It should be noted that this trend may indicate two opposing processes: an improvement in disease diagnosis due to the gradual development of preventive medicine and dispensary monitoring of public health, while at the same time, a deterioration in healthcare in general due to the Russian healthcare reforms aimed at optimising inefficient spending by closing inefficient hospitals and expanding the use of high technology. This eventually resulted in the following (Compiled from: Regions of Russia. Socioeconomic indicators, 2022. [Electronic resource]: https://rosstat.gov.ru/folder/210/document/13204 (accessed on15 January 2023)):

1. The number of beds decreased from 111.0 per 10,000 people in 2005 to 79.8 in 2021, with more beds required after the COVID-19 pandemic, and the number of beds is continuing to decrease.
2. The number of nursing staff decreased from 108.0 per 10,000 people in 2005 to 100.8 in 2021.
3. The number of visits per 10,000 people in outpatient clinics increased from 256.0 in 2005 to 292.2 in 2021.
4. There was a slight increase in the number of doctors, from 45.9 per 10,000 people in 2005 to 48.8 in 2021.

Reforms in the healthcare system have led to a reduction in the availability of inpatient care and an increase in the availability of outpatient care, while its commercialisation has also increased. Critics of this reform note that access to specialist care has declined sharply and that the availability of medical care has decreased, particularly in remote areas [40].

## 5. Conclusions

Our hypothesis—that the factors determining the socioeconomic situation in the country contribute to the level and dynamics of mortality of the working-age population, but to different degrees and in different directions depending on the nature of the socioeconomic changes taking place in specific periods—has been confirmed. Applying the methods of machine learning and data mining, we found that in each time period determined for the study, socioeconomic factors act on the mortality rate with different strengths and directions, creating both positive and negative vectors of influence on its dynamics. A significant impact on the growth of mortality of the working-age population is caused by factors of life safety, reflected first of all in indicators of crime rate, as well as factors that characterise working conditions, determining the level of injuries at work, including fatalities and occupational diseases of workers. The healthcare system in the period under study went through difficult times related to reforming and optimising the parameters of its functioning, which led to its generally insignificant contribution to the reduction in mortality in the working-age population.

Therefore, the further development of methodological tools to measure the partial contribution of socioeconomic factors to the level and dynamics of mortality of the working-age population, based on machine learning methods, is an important area of research. It contributes to a better understanding of the mechanism of the formation of cause–effect relationships between various factors and public health. It leads to the conviction that a comprehensive approach to the development of public health policy should take into account not only measures to promote health, but also measures to improve working conditions and growth and the social wellbeing of the population.

## Figures and Tables

**Table 1:** Average Pearson correlation coefficients between working-age mortality and indicators of socioeconomic development of Russian regions, 2005–2021.

Index	Correlation Coefficient	Index	Correlation Coefficient
Number of reported crimes per 100,000 people	0.614	Budget expenditure per capita, thousand RUB	0.216
Number of hospital beds per 10,000 people	0.599	Number of private cars per 1000 people, units	0.133
Outpatient polyclinic capacity, visits per shift per 10,000 people	0.544	Ratio of average monthly wage to subsistence minimum, times	0.079
Share of workers in hazardous and dangerous jobs (by category of hazard), % of total economic employment	0.432	Share of budget expenditure on social assistance, %	0.060
Nursing staff availability per 10,000 people	0.409	Total unemployment rate, %	−0.390
Share of household expenditure on alcoholic beverages, %	0.389	Density of paved public roads, km of track per 10,000 km^2^ of territory	−0.419
Total illness rate per 1000 people	0.338	Share of employed population with higher education, %	−0.449
Share of industry in the GVA of Russian regions, %	0.291		

**Table 2:** Clustering indicators to assess the partial contribution of factors to working-age mortality.

Factor Block	Indices
Working conditions	Share of workers in hazardous and dangerous jobs, % Share of employed population with higher education, % Total unemployment rate, % Share of industry in the GVA of Russian regions, %
State of the healthcare system	Outpatient polyclinic capacity, visits per shift per 10,000 people Total illness rate per 1000 people Nursing staff availability per 10,000 people Occupational illnesses (number of employees diagnosed for the first time), persons
Life safety	Number of reported crimes per 100,000 people Density of paved public roads, km of track per 10,000 km^2^ of territory Share of household expenditure on alcoholic beverages, %
Living standards	Budget expenditure per capita, thousand RUB Share of budget expenditure on social assistance, % Number of private cars per 1000 people, units Wage purchasing power (ratio of average monthly wage to subsistence minimum) times

## Data Availability

Not applicable.
